# Evolutionary potential in the Alpine: trait heritabilities and performance variation of the dwarf willow *Salix herbacea* from different elevations and microhabitats

**DOI:** 10.1002/ece3.2171

**Published:** 2016-05-12

**Authors:** Janosch Sedlacek, Andrés J. Cortés, Julia Wheeler, Oliver Bossdorf, Guenter Hoch, Jaroslav Klápště, Christian Lexer, Christian Rixen, Sonja Wipf, Sophie Karrenberg, Mark van Kleunen

**Affiliations:** ^1^ EcologyDepartment of BiologyUniversity of KonstanzUniversitätsstraße 1078457KonstanzGermany; ^2^ Department of Ecology and GeneticsUppsala UniversityNorbyvägen 18 D75236UppsalaSweden; ^3^ WSL Institute for Snow and Avalanche Research SLFFlüelastrasse 117260DavosSwitzerland; ^4^ Institute of BotanyUniversity of BaselSchönbeinstrasse 64056BaselSwitzerland; ^5^ Plant Evolutionary EcologyInstitute of Evolution and EcologyAuf der Morgenstelle 5University of Tübingen72076TübingenGermany; ^6^ Department of Forest and Conservation SciencesFaculty of ForestryUniversity of British Columbia2424 Main MallVancouverBritish ColumbiaV6T 1Z4Canada; ^7^ Department of Genetics and Physiology of Forest TreesFaculty of Forestry and Wood SciencesCzech University of Life Sciences in PragueKamýcká 129165 21Prague 6Czech Republic; ^8^ Department of Botany and Biodiversity ResearchUniversity of ViennaRennweg 14A‐1030ViennaAustria; ^9^ Present address: Department of Plant BiologySwedish Agricultural UniversityUndervisningsplan 7E75007UppsalaSweden; ^10^ Present address: Department of Environmental ConservationUniversity of MassachusettsAmherstMassachusetts01003; ^11^ Present address: Scion (New Zealand Forest Research Institute Ltd.)49 Sala StreetWhakarewarewa3046RotoruaNew Zealand

**Keywords:** Adaptive evolution, alpine ecosystem, animal model, long‐lived plants, snowmelt microhabitats, SSR markers

## Abstract

Alpine ecosystems are seriously threatened by climate change. One of the key mechanisms by which plants can adapt to changing environmental conditions is through evolutionary change. However, we still know little about the evolutionary potential in wild populations of long‐lived alpine plants. Here, we investigated heritabilities of phenological traits, leaf size, and performance traits in natural populations of the long‐lived alpine dwarf shrub *Salix herbacea* using relatedness estimates inferred from SSR (Simple Sequence Repeat) markers. *Salix herbacea* occurs in early‐ and late‐snowmelt microhabitats (ridges and snowbeds), and we assessed how performance consequences of phenological traits and leaf size differ between these microhabitats in order to infer potential for evolutionary responses. *Salix herbacea* showed low, but significant, heritabilities of leaf size, clonal and sexual reproduction, and moderate heritabilities of phenological traits. In both microhabitats, we found that larger leaves, longer intervals between snowmelt and leaf expansion, and longer GDD (growing‐degree days) until leaf expansion resulted in a stronger increase in the number of stems (clonal reproduction). In snowbeds, clonal reproduction increased with a shorter GDD until flowering, while the opposite was found on ridges. Furthermore, the proportion of flowering stems increased with GDD until flowering in both microhabitats. Our results suggest that the presence of significant heritable variation in morphology and phenology might help *S. herbacea* to adapt to changing environmental conditions. However, it remains to be seen if the rate of such an evolutionary response can keep pace with the rapid rate of climate change.

## Introduction

Snowmelt patterns in alpine ecosystems are considerably impacted by current changes in temperature and precipitation. Earlier snowmelt may extend the growing season above altitudes of 2000 m by up to 60 days (Beniston et al. 2003). This is likely to have strong impacts on plants by altering the developmental timing, increasing exposure to frost, changing moisture availability in the soil and affecting interactions with other plants, pollinators, herbivores, and pathogens (Beniston et al. 2003; Inouye 2008; Wipf et al. 2009; Little 2014; Wheeler et al. 2014). Therefore, predicting responses of species to climate change is one of the most pressing challenges in current ecological research (Theurillat and Guisan 2001; Walther et al. 2002; Bellard et al. 2012).

Most research on responses of alpine plant species to changing snowmelt and temperature conditions has focused on species migration toward higher altitudes, as this may allow plants to track their climatic requirements (Conradin and Walther 2005; Lenoir et al. 2008; Eskelinen et al. 2009; Matteodo et al. 2013; Wipf et al. 2013). However, if migration potential is limited (Jump and Penuelas 2005; Aitken et al. 2008; Sedlacek et al. 2014), the only way plants can persist is by adjusting to the new environmental conditions. Adjustment through phenotypic plasticity might be particularly important in long‐lived species, as plastic responses can occur within the lifetime of an individual (Nicotra et al. 2010). Plastic responses to warmer temperatures have been demonstrated for changes in phenology (e.g., in timing of bud burst; Kramer 1995; Anderson et al. 2012; Sedlacek et al. 2015). However, plasticity may be constrained or even maladaptive, if species or populations are exposed to novel environmental conditions outside the range of conditions they encountered in their evolutionary history (Ghalambor et al. 2007; Visser 2008; Scheepens and Stöcklin 2013).

Another way organisms can adjust to climate change is through adaptive evolution, which could be a key mechanism in long‐term responses to climate change. However, it is unclear whether populations have enough evolutionary potential to allow them to keep up with the pace of climate change (Bradshaw and Holzapfel 2006; Visser 2008; Hoffmann and Sgrò 2011; Franks et al. 2014). There is considerable evidence that evolutionary responses can indeed occur on a contemporary time scale (reviewed by Franks et al. 2014), whereas other studies suggest that evolutionary responses may be too slow to allow for adaptation to future climates (e.g., Billington and Pelham 1991; Anderson et al. 2012). Nonetheless, the number of empirical studies that have estimated the potential for evolutionary response of plants is still limited, particularly for long‐lived species and under natural field conditions (but see: van Kleunen and Ritland 2004).

To estimate evolutionary responses, both heritability and consequences of trait variation for fitness need to be estimated (Lande and Arnold 1983; Falconer and Mackay 1996). Different traits may also be genetically correlated, and these genetic correlations may inhibit or reinforce evolutionary change (Falconer and Mackay 1996). The heritability and fitness consequences of, and genetic correlations among traits may all vary among environments (Wilson et al. 2006; Husby et al. 2011). Studies that estimate heritability and fitness consequences under artificial conditions might thus not be representative of in situ circumstances. Therefore, it is important to study the potential for adaptive evolution of several, potentially correlated, traits in natural populations under a range of environmental conditions (Kruuk et al. 2008), even though measures of both relatedness and fitness or performance can be challenging.

To assess which functional traits are under selection, and how this depends on the environment, one has to assess the effects of these traits on fitness (e.g., Lande and Arnold 1983). Fitness refers to the demographic contribution of a genotype to the next generation (de Jong 1994). In short‐lived plants, fitness is often estimated as survival and/or total seed production, whereas in long‐lived species estimating fitness can be more difficult. In such cases, analyses using performance indicators such as growth or measures of flower number, which are expected to be correlated with true fitness, may be the only option. Clonal plants present an additional difficulty. Although sexual reproduction is most intimately linked to fitness, it can be rare in some clonal species, particularly in alpine habitats (Chambers 1995; Forbis 2003). Clonal reproduction on the other hand, affects survival and expansion of the genet (and thereby future sexual reproduction), might trade‐off with sexual reproduction (Watson and Casper 1984; Prati and Schmid 2000; van Kleunen et al. 2002), and might enhance sexual reproduction by increasing pollen and seed dispersal (Van Drunen et al. 2015). As a consequence, it is unclear how sexual and clonal reproduction could be integrated into one performance or fitness measure, and they are therefore often treated separately.

Estimating heritability requires a known pedigree or marker‐inferred pairwise relatedness between individuals, and quantitative trait values of these individuals. One powerful approach for estimating heritabilities in the wild is to use these pairwise relatedness estimates in so‐called animal‐model analyses (Milner et al. 2000; Frentiu et al. 2008; Bérénos et al. 2014; Klápště et al. 2014). Animal‐model analyses are linear mixed models that can be used to estimate the additive genetic contribution to phenotypic trait variation and thus trait heritability (Wilson et al. 2010). Moreover, such animal‐model analyses can account for nongenetic sources of variation (e.g., microhabitats) by including these sources as fixed or random effects (Wilson et al. 2010).

Despite considerable progress in the development of methods for inferring quantitative genetic parameters in natural populations, only a few studies have estimated heritabilities in long‐lived plant species using molecular markers (Klaper et al. 2001; Ritland and Travis 2004; Andrew et al. 2005; Blows and Hoffmann 2005; Herrera and Bazaga 2009; Klápště et al. 2014). On mountain slopes, temperature, snowmelt timing and competition, which are important selective pressures, have been demonstrated to vary with altitude and among microhabitats (Körner 2007; Scherrer and Körner 2011). The same environmental factors, temperature, and snowmelt timing, are expected to change under climate warming (Körner 2007). Here, we exploit this situation and investigate the potential for adaptation to current small‐scale environmental heterogeneity in the long‐lived arctic‐alpine dwarf shrub, *Salix herbacea*, in order to contribute to the understanding of the species' potential to respond to changing climatic conditions.

Previously, we showed in a 3‐year observational study that most trait variation in *S. herbacea* is associated with variation in snowmelt timing (Wheeler et al. 2016). In the current study, we investigate the potential for adaptive evolution of *S. herbacea* growing in early‐ and late‐snowmelt microhabitats. We assessed narrow‐sense heritability (*h* ^2^) and genetic covariance of phenological traits (i.e., snowmelt‐to‐leaf‐expansion interval, GDD [growing‐degree days] until leaf expansion, GDD until flowering) and leaf size, as well as of traits related to clonal reproduction (relative change in stem number) and to sexual reproduction (proportion of flowering stems). Moreover, we analyzed whether variation in phenological traits and leaf size is associated with clonal or sexual reproduction.

## Methods

### Study species

*Salix herbacea* is a dioecious, long‐lived clonal dwarf shrub with an alpine‐arctic distribution. It is widespread and common in the northern and alpine regions of Europe and North America, and in western Siberia and the Arctic region (Beerling 1998). *Salix herbacea* is common in snowbeds and also occurs on wind‐exposed mountain ridges and screes, where snow cover disappears earlier in the spring (Beerling 1998). *Salix herbacea* reproduces both sexually and clonally, the latter by producing an extensive ramifying system with branched rhizomes (Beerling 1998). *Salix herbacea* may produce >4000 seeds/m^2^ (Nyléhn et al. 2000), and seeds are wind‐dispersed. The aerial branches (ramets) are woody and usually reach only 2–5 cm above the ground. Most clones of *S. herbacea* at our study sites were smaller than 20 cm in diameter, however, clones with diameters up to several meters have also been observed, both at our study sites (Häggberg, Cortés and Karrenberg, unpublished data) and in other populations (Reisch et al. 2007; de Witte et al. 2012).

### Measurements of phenotypic traits and temperatures

In the summer of 2011, we sampled *S. herbacea* patches at a total of 12 sites, distributed across three mountains (Jakobshorn, Schwarzhorn, and Wannengrat, Table S1) in the Swiss Alps near Davos (46° 48′ N 9° 49′ E). To cover the altitudinal distribution and microhabitat range of *S. herbacea*, we selected four sites at each mountain: one ridge and one snowbed site (i.e., an early‐ and a late‐snowmelt site, respectively) in the higher part of the elevational range (2414–2768 m asl) and one ridge and one snowbed site in the lower part of the elevational range (2180–2352 m asl). The identification of ridge and snowbed microhabitats was based on topology and vegetation (Odland and Munkejord 2008; Sedlacek et al. 2015), and microhabitat sites differed significantly in snowmelt timing (Table S2, see below for a description of measurements of snowmelt timing). The average (±SE) distance between sites on each mountain was 867 ± 291 m, and the average site area was 300 ± 49 m^2^. At each site, we marked between 82 to 94 *S. herbacea* patches with a diameter of 10 cm that were more than 1 m apart, yielding a total of 1061 *S. herbacea* patches across the four sites on each of the three mountains.

During the 2011 and 2012 growing seasons, we monitored phenology and the proportion of flowering stems of all *S. herbacea* patches weekly from snowmelt until leaf senescence. Intervals from snowmelt to each phenophase (leaf expansion, flowering, fruiting) were calculated as the difference from day of snowmelt until first occurrence of the respective phenophase. Further, we recorded the stem density (i.e., number of stems per 10‐cm diameter patch) and the mean area of a leaf (i.e., *π *× ½ length* *× ½ width taken from two undamaged leaves) for each patch. We measured soil temperature at 5 cm below the surface at 2‐h intervals, using five in situ soil temperature loggers (iButton, Maxim Integrated, San Jose, CA) per site. The temperature data, as well as field observations, were then used to determine the day of snowmelt (the day when soil temperature rose sharply from near zero °C, which characterizes insulation by snow cover; Wheeler et al. 2014). We also used the temperature data to calculate GDD above 5°C, as GDD based on this threshold shows the best correlation with growth of alpine and arctic plants (Molau and Mølgaard 1996). The GDD until each phenophase was calculated as GDD accumulation from snowmelt day to the first occurrence of each phenophase.

In clonal plants, like *S. herbacea*, both clonal reproduction and sexual reproduction contribute to fitness and performance. In the ideal case, one would assess absolute performance measures per genet, such as the total numbers of flowers and of ramets per genet. However, as it was not possible to identify entire genets of *S. herbacea*, which can be several meters in diameter (Reisch et al. 2007; de Witte et al. 2012), we focused on patches of the *S. herbacea* genets. As these patches differed in stem density, we measured clonal reproduction as the ratio of stem number in 2012 to stem number in 2011 (i.e., as the relative change in stem number), and sexual reproduction as the proportion of flowering stems recorded in 2012. We used flowering stems in 2012 instead of 2011 because flower buds are preformed in the previous year. Although these are relative performance measures, they should give a good indication of the long‐term performance of the patches and the genets they belong to.

### SSR‐marker analysis

Between July and August 2011, we sampled five leaves from a single stem per patch, immediately stored them in tea bags and dried them in a plastic container with silica gel (Rubin; Sigma Aldrich, Munich, Germany). Genomic DNA was extracted using the QIAGEN DNeasy 96 Plant Kit (QIAGEN, Hilden, Germany) following the manufacturer's instructions. DNA concentration and purity was quantified using a NanoDrop spectrophotometer ND‐1000 (NanoDrop Technologies, Wilmington, DE), and samples were stored at −18°C. The primers for the seven SSR loci used in this study were also used in a previous study of *S. herbacea* (Cortés et al. 2014). The PCR reactions were multiplexed in two PCR runs (Table S3) using the QIAGEN Multiplex PCR Kit (QIAGEN) following the manufacturer's instructions. The PCR products were pooled and separated by capillary electrophoresis at Uppsala University, Uppsala, Sweden, using an ABI 3130 DNA Analyzer and LIZ500 as ladder (Applied Biosystems, Foster City, CA). We estimated allele sizes in base‐pairs using GeneMapper v.3.7 (Applied Biosystems). On average, we found 23 alleles per SSR locus. For information on allele frequencies, heterozygosity, effective number of alleles, estimated null allele frequencies and deviations from Hardy–Weinberg equilibrium (HWE), see Table S3. These statistics were computed with GENEPOP v.3.5 (Raymond and Rousset 1995), and suggest that there are no unusual patterns of variation that require removal of certain markers.

### Relatedness estimation using SSR genotypes

Any combination of at least five of our SSR markers was enough to reduce the probability of identity of any two different genotypes close to zero (Fig. S1). Among the 1061 sampled patches, we identified 939 unique multilocus SSR genotypes. These were identified and assigned using the Lynch distance (Lynch 1990) with a threshold of 0.05 in the R package *polysat* (Clark and Jasieniuk 2011). Lynch distances below 0.05 were interpreted as identical genotypes based on the abundance of pairwise comparisons with very low genetic distance. Keeping these presumed clonal replicates in the dataset would result in several rows and columns of the genetic‐distance matrix having identical values, which would cause problems for inversion of the matrix to obtain the relatedness matrix. To avoid singularity in the relatedness matrix (see below), we kept only one randomly selected patch per genotype in the dataset. Because the performance of different relatedness estimators is still under discussion (Van de Casteele et al. 2001; Csilléry et al. 2006), we calculated relatedness matrices using four different pairwise relatedness estimators (Queller and Goodnight 1989; Li et al. 1993; Lynch and Ritland 1999; Wang 2002), implemented in SPaGeDi v. 1.4 software (Hardy and Vekemans 2002). Diagonal elements of the matrices were set to one as they describe the relatedness of a genotype with itself. The nearest positive definite matrix of the relatedness matrix was computed using the *nearPD* function in the R package *Matrix* (Bates and Maechler 2015; see also Klápště et al. 2014). For a summary of relatedness estimates between the 440,391 pairwise comparisons (939 patches) using the four estimators, see Table S4. In the main text, we only report the results on heritability values obtained using the Lynch and Ritland (1999) relatedness estimator because this is the most commonly used estimator and thus makes comparisons with other studies easier. The results obtained using the other estimators can be found in the Supporting information (Tables S5 and S7–S9), and correlated well with the ones of the Lynch and Ritland estimator (Pearson correlations between heritabilities ranged from 0.71 to 0.95; Table S5).

### Estimation of genetic parameters and breeding values

Variance and covariance components of the ***G*** matrix and narrow‐sense heritabilities (*h* ^2^) of phenological traits (snowmelt‐to‐leaf‐expansion interval, GDD until leaf expansion, GDD until flowering) and leaf size, measured in 2011, and of fitness traits (change in stem number, proportion of flowering stems) were estimated across all sites with the multivariate REML animal model (Kruuk 2004). This analysis, implemented in ASReml‐R v.3.0 (Butler et al. 2007), estimates an individual′s breeding value (i.e., additive genotype) and treats it as a random effect in order to partition the total phenotypic variance into additive genetic variance and residual variance, as

(1) $$y_{i}=X\beta_{i}+Za_{i}+e_{i}$$

Here **y**_***i***_ is the vector of observed phenotypic values of the *i*th trait, **β**_*i*_ the vector of fixed effects of the *i*th trait, **a**_*i*_ the vector of additive genetic effects (individual's breeding values) of the *i*th trait, **e**_*i*_ the vector of residual effects for the *i*th trait, and ***X*** and ***Z*** are incidence matrices relating fixed and random effects to values in vector **y**_*i*_. By estimating the vector of additive genetic effects, the additive genetic variance can be calculated as $\text{Var}\left(a_{i}\right)=M\;*\sigma_{a_{i}}^{2}$, where $\sigma_{a_{i}}^{2}$ is the additive genetic variance of the *i*th trait and ***M*** is the SSR‐inferred relatedness matrix. Similarly, by estimating the vector of residual effects, the residual variance can be calculated as $\text{Var}\left(e_{i}\right)=I\sigma_{e_{i}}^{2}$, where $\sigma_{e_{i}}^{2}$ is the residual variance of the *i*th trait and ***I*** is an identity matrix.

In the study region, gene flow in *S. herbacea*, within and between mountains, is very high, as the number of migrants per generation across mountains was estimated as 1.6 ± 0.1 (Cortés et al. 2014). Therefore, we consider all 12 study sites to be part of one big population, and we consequently performed the heritability analysis using all 870 patches with complete trait data. Cortés et al. (2014) found no local genetic structure in *S. herbacea* in the study region, but they found an indication for isolation by distance, which caused a deviation from HWE. We accounted for isolation by distance and for environmental variation between the sites (Wilson et al. 2010) by using transect, elevation, microhabitat, and the interactions between microhabitat and elevation and between microhabitat and transect as fixed terms in the model. We did not include the elevation* *× transect and microhabitat* *× elevation* *× transect interactions in the final model because models with these interactions did not converge, most likely due to missing data at one of the sites.

The narrow‐sense heritability (*h* ^2^) of the *i*th trait was estimated as the ratio of the additive genetic variance to the total phenotypic variance (Falconer and Mackay 1996):

(2) $${{\hat{h}}_{i}}^{2}=\frac{{{\hat{\sigma }}^{2}}_{a_{i}}}{{{\hat{\sigma }}^{2}}_{a_{i}}+{{\hat{\sigma }}^{2}}_{e_{i}}}$$

where ${\hat{\sigma }}_{a_{i}}^{2}$ and ${\hat{\sigma }}_{e_{i}}^{2}$ are estimates of the additive genetic and residual variance of the *i*th trait, respectively. Confidence intervals of ${\hat{h}}_{i}^{2}$ were calculated using the *pin* function implemented in the R package *nadiv* (Wolak 2012). *Z*‐tests and Wald *F*‐tests were used to assess the significance of variance components and fixed terms, both implemented in ASReml‐R (Butler et al. 2007).

### Effects of phenological traits and leaf size on performance

To test whether phenological and morphological traits affect performance, we fitted the relative change in stem number (related to clonal reproduction) and the proportion of flowering stems (related to sexual reproduction) against the standardized phenotypic traits using multiple regression with linear mixed models as implemented in the *nlme* package (Pinheiro et al. 2014). Standardization allows for comparisons between traits similar to a selection‐gradient analysis (Lande and Arnold 1983). Our analysis does not represent a formal selection‐gradient analysis as we do not have fitness proxies representing a demographic contribution to the next generation. We included microhabitat and the interactions between traits and microhabitat in the regression models, as performance can be affected differently by the traits in the two microhabitats. Initially, we also included elevation and interactions between traits and elevation, and interactions among traits, elevation and microhabitat in the model. However, as we neither found significant differences in day of snowmelt between the low and high elevations (Wheeler et al. 2014) nor differences in fitness consequences of the traits between them, elevation was dropped from the final model. If the trait* *× microhabitat interaction was significant, we used separate regression analyses for ridge and snowbed microhabitats for further analysis of the respective traits. To account for nonindependence of patches measured in the same site and on the same mountain, we included site nested within transect and transect as random effects. All statistical analyses were done using R version 2.15.2 (R Core Team 2012).

## Results

### Heritability estimates

The multivariate form of the animal model revealed significant estimates of narrow‐sense heritability (${\hat{h}}^{2}$) for all measured traits, with the exception of the proportion of flowering stems (Table 1). For leaf size (${\hat{h}}^{2}$ = 0.061) and the change in stem number (${\hat{h}}^{2}$ = 0.071), the *h* ^2^ estimates were relatively low. For the snowmelt‐to‐leaf‐expansion interval (${\hat{h}}^{2}$ = 0.178), and GDD until leaf expansion (${\hat{h}}^{2}$ = 0.141) and GDD until flowering (${\hat{h}}^{2}$ = 0.181), the *h* ^2^ estimates were moderate. The magnitude and the significance of these *h* ^2^ estimates based on the Lynch and Ritland relatedness estimator were largely consistent with those obtained using the other three relatedness estimators (Table S5).

**Table 1:** Estimates of narrow‐sense heritability (*h* ^2^) and its 95% confidence intervals (lowCI, upCI), and estimates of the additive genetic variance (Va) and the residual variance (Vr) for morphological (leaf size), performance (change in stem number, proportion of flowering stems) and phenological (snowmelt‐to‐leaf‐expansion interval, growing‐degree days [GDD] until leaf expansion, GDD until flowering) traits. Analysis were done using the multivariate animal model with a SSR‐computed relatedness matrix (Lynch and Ritland 1999). For estimates obtained by using the Wang (2002), Queller and Goodnight (1989) and Li et al. (1993) relatedness estimators, see Table S5

Trait	*h* ^2^	lowCI	upCI	Va	Vr
Leaf size	**0.061**	0.011	0.112	**0.128**	**1.952**
Change in stem number	**0.071**	0.014	0.128	**0.016**	**0.207**
Proportion of flowering stems	0.034	−0.018	0.087	0.001	0.036
Snowmelt‐to‐leaf‐expansion interval	**0.178**	0.111	0.245	**10.360**	**47.842**
GDD until leaf expansion	**0.141**	0.008	0.275	**0.109**	**0.664**
GDD until flowering	**0.181**	0.032	0.330	**0.155**	**0.700**

Significant values are in bold.

### Performance consequences of variation in phenological and morphological traits

**Figure 1:**
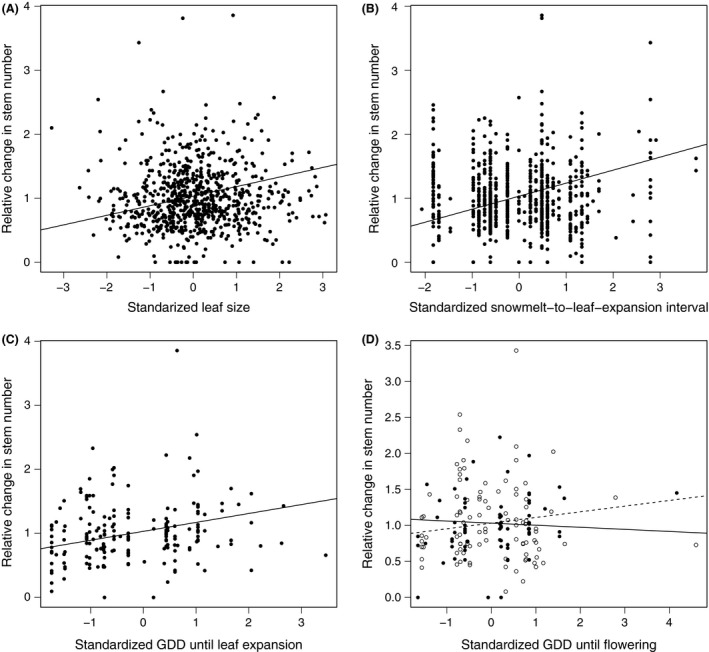
Significant linear regressions of relative clonal reproductive fitness (measured as the change in stem number) on standardized (A) leaf size, (B) snowmelt‐to‐leaf expansion interval, (C) growing‐degree days (GDD) until leaf expansion and (D) GDD until flowering. In d, the association between change in stem numbers and GDD until flowering differed between ridge sites (black symbols and solid line) and snowbed sites (white symbols and dotted line).

**Figure 2:**
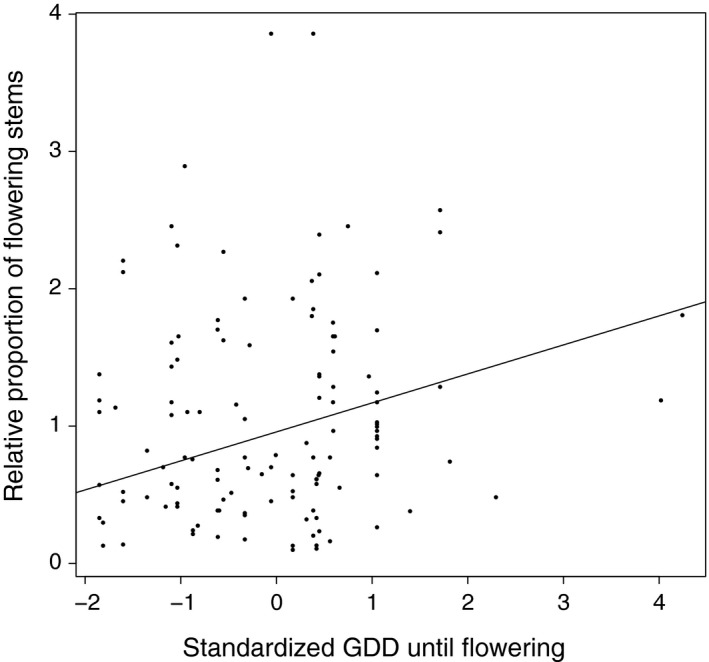
Significant linear regression of the relative proportion of flowering stems, as a proxy for sexual reproductive fitness, on standardized growing‐degree days until flowering.

The relative change in stem number (clonal reproduction) increased significantly with increasing leaf size (Fig. 1A) and increasing GDD until leaf expansion (Table 2, Fig. 1C), and marginally so with a longer snowmelt‐to‐leaf‐expansion interval (Table 2, Fig 1B). These relationships were the same for both microhabitats (no significant trait × microhabitat interaction, Table 2). Relative change in stem number was also affected by GDD until flowering, but the direction of this effect depended on the microhabitat type (significant trait* *× microhabitat interaction, Table 2, Fig. 1D). Relative change in stem number increased with longer GDD until flowering on ridges (Table S6), and with shorter GDD until flowering in snowbeds (Table S6). The proportion of flowering stems (sexual reproduction) increased significantly with increasing GDD until flowering, both in the ridge and snowbed microhabitat types (Table 2, Fig. 2).

**Table 2:** Effects of leaf size, snowmelt‐to‐leaf‐expansion interval, growing‐degree days (GDD) until leaf expansion and GDD until flowering, and their interactions with microhabitat type on the change in stem number (upper part of table) and the proportion of flowering stems (lower part of table). The estimates (Est.) are from linear mixed models in which plot was nested within transect as random effect. When the interaction of a trait with microhabitat type was significant, we used estimates of the coefficients for ridge and snowbed microhabitats separately (see Table S6 for separate analyses of ridge and snowbed microhabitats)

Relative fitness	Trait (standardized) and interaction	Est.	df	*F*	*P*
Change in stem number	Leaf size	0.149	103	3.574	**0.041**
	Snowmelt‐to‐leaf‐expansion interval	0.203	103	1.935	**0.046**
	GDD until leaf expansion	0.139	103	4.703	**0.032**
	GDD until flowering	−0.029	103	0.002	0.967
	Microhabitat* *× Leaf size	−0.073	103	0.454	0.501
	Microhabitat* *× Snowmelt‐to‐leaf‐expansion interval	−0.283	103	6.888	**0.010**
	Microhabitat* *× GDD until leaf expansion	−0.087	103	0.647	0.423
	Microhabitat* *× GDD until flowering	0.103	103	1.295	**0.048**
Proportion flowering stems	Leaf size	−0.023	66	0.032	0.859
	Snowmelt‐to‐leaf‐expansion interval	−0.029	66	0.342	0.561
	GDD until leaf expansion	−0.041	66	0.426	0.516
	GDD until flowering	0.211	66	4.153	**0.046**
	Microhabitat* *× Leaf size	0.054	66	0.076	0.784
	Microhabitat* *× Snowmelt‐to‐leaf‐expansion interval	0.179	66	1.110	0.296
	Microhabitat* *× GDD until leaf expansion	−0.095	66	0.376	0.542
	Microhabitat* *× GDD until flowering	−0.074	66	0.163	0.688

Significant values are in bold.

## Discussion

### Heritability estimation in the wild

Using the animal model with SSR‐based relatedness estimates in natural populations of *S. herbacea*, we found low to moderate heritabilities for phenological traits, leaf size and clonal reproduction. In situ estimation of heritability is conceptually and computationally challenging, and such estimates are rarely reported for plants (for wild animal populations, see e.g., Husby et al. 2011; Réale et al. 2003). So, despite the large number of studies on selective forces in wild populations (reviewed by Kingsolver and Diamond 2011), our knowledge about the evolutionary potential in wild plant populations is still limited.

The performance of different relatedness estimators for calculating heritabilities has been shown to largely depend on the breeding system, population and relatedness structure, sample size, the number of molecular markers and the pattern of variation in the molecular markers (Van de Casteele et al. 2001; Ritland and Travis 2004; Coltman 2005; Rodríguez‐Ramilo et al. 2007; Bessega et al. 2009; Korecký et al. 2014). Although some studies suggested that large numbers of markers are required for accurate relatedness estimates (Wilson et al. 2003; Coltman 2005), Bessega et al. (2009) showed in a study on an experimental stand of the tree *Prosopis alba* that heritabilities based on relatedness inferred from six SSR loci were highly correlated (average *R* ^2^ = 0.57) to those based on replicated half‐sib families. This suggests that the seven highly variable SSR markers in our study were sufficient for inferring relatedness in our study. SSR markers are highly polymorphic and offer good resolution for distinguishing genotypes and different levels of relatedness among individuals (Ellegren 2004). Furthermore, the consistency of the estimates of the additive genetic and residual variance components, and consequently of the heritability estimates, among the four relatedness estimators (Table S5) suggests that our heritability estimates are robust with regard to the relatedness estimator.

The majority of heritability estimates in wild populations are for vertebrate species. In a review of more than 1500 heritabilities, estimated with various methods, in wild vertebrate populations, Postma (2014) reports average values of 0.56 and 0.33 for morphological and life‐history traits, respectively. These values are higher than the values that we found for traits of *S. herbacea*. However, Postma (2014) also found significantly lower heritability estimates when the animal model was used instead of other approaches. A possible explanation for this is that the animal model usually accounts for other sources of similarity between relatives, for example due to shared environments, which are ignored in other approaches (Postma 2014). In addition, marker‐based estimates have been reported to be biased downward compared to estimates based on traditional half‐sib designs (Thomas and Hill 2000; van Kleunen and Ritland 2005; Gay et al. 2013). Therefore, it is likely that our calculated heritabilities underestimate the true heritabilities.

We detected significant heritabilities for phenological traits and leaf size in *S. herbacea,* suggesting genetically based traits variation that potentially allows for responses to selection. However, heritability values were of low to moderate magnitude for phenological traits and of low magnitude for leaf size, suggesting that a large proportion of the trait variation is caused by environmental or developmental variation. A strong environmental effect on phenology in wild populations is supported by a previous reciprocal transplant study, where the phenology of *S. herbacea* demonstrated a strong plastic response to snowmelt timing (Sedlacek et al. 2015). In greenhouse studies, however, where environmental variation is reduced, comparatively high heritability values for phenological traits in the range of 0.5–0.9 have been reported (Yao and Mehlenbacher 2000; Keller et al. 2011; Frei et al. 2014). Apart from phenotypic plascticity, low heritability could also be the result of strong selection in the past that has depleted genetic variation and this may be true for traits closely related to performance in our study such as the change in stem number and the proportion of flowering stems (Falconer and Mackay 1996).

### Performance consequences of variation in phenology and leaf size

Even though our study does not allow for a formal selection‐gradient analysis, associations between heritable traits and clonal or sexual reproduction can suggest the direction of possible selection pressures and evolutionary responses. Our interpretations are, like most studies, limited by the comparatively short duration of our study, which excludes effects of between‐year climatic variation or extreme events. We found that leaf size, snowmelt‐to‐leaf‐expansion interval and GDD until leaf expansion, all had significant heritabilities, and were positively associated with clonal reproduction (estimated as the relative change in the number of stems). This suggests that the values of these morphological and phenological traits may increase in the population as a whole. Possible selective pressures for later leaf expansion could be related to frost avoidance early in spring (Wheeler et al. 2014), whereas plants with larger leaves may have higher photosynthetic productivity (Smith and Geller 1979). GDD to flowering had a moderate heritability, and affected clonal reproduction positively in ridge but negatively in snowbed microhabitats. At the same time, GDD to flowering was positively associated with sexual reproduction estimated as the proportion of flowering stems in both microhabitats. A rapid phenology might be especially important in snowbeds where *S. herbacea* must start flowering and fruiting shortly after snowmelt in order to produce seeds before the first autumn frost and snowfall, and this possibly leads to selection for earlier flowering in these microhabitats. On ridges, in contrast, plants have a higher risk to suffer from early‐spring frost events and increased insect herbivory (Wheeler et al. 2014; Sedlacek et al. 2015), possibly leading to opposing selection for later flowering. Stinson (2004) found the same pattern of opposing performance consequences of flowering phenology in a long‐lived subalpine plant at opposite ends of a snowmelt gradient, and argued that physiological stresses under early snowmelt may slow down reproduction. In a recent meta‐analysis, selection was found to favor earlier flowering individuals in 87 plant species, particularly at high latitudes, where growing seasons are short (Munguía‐Rosas et al. 2011; see also Anderson et al. 2012). Our data are consistent with divergent selection on flowering time in the two microhabitats in *S. herbacea*, but an ongoing response to such divergent selection is unlikely because of the high gene flow between subpopulations in different microhabitats (Cortés et al. 2014).

In a climate‐warming scenario, snowmelt is likely to occur earlier, like on ridges today. Although snowbeds and ridges do not only differ in snowmelt timing, the difference in the relationship of clonal reproduction with GDD to flowering between both microhabitat types (Fig. 1D) suggests that selection for increases in GDD to flowering could become stronger in the future. However, as the positive relationships of clonal reproduction with leafing phenology and leaf size were not different between snowbeds and ridges (Fig. 1A–C), selection regimes on these traits are not likely to change under a future climate. Divergent selective forces on GDD until flowering are also likely to become stronger under ongoing climate change (Frei et al. 2014; Gienapp et al. 2014). Adaptive evolution may be constrained by antagonistic genetic correlations, which can be affected by climate change (Etterson and Shaw 2001; Garant et al. 2008; Walsh and Blows 2009). However, under the current conditions, the genetic covariance components among the traits in our study were mostly nonsignificant (Table 3). In other words, there was no evidence that evolutionary responses could be constrained by trait correlations.

**Table 3:** The matrix of additive genetic variances and covariances (G matrix) using the Lynch and Ritland (1999) relatedness estimator (see Table S7–S9 for the other estimators)

Trait	(1)	(2)	(3)	(4)	(5)	(6)
(1) Leaf size	**0.128**					
(2) Change in stem number	0.020	**0.016**				
(3) Proportion of flowering stems	0.008	0.002	0.001			
(4) Snowmelt‐to‐leaf‐expansion interval	−0.079	−0.034	−0.084	**10.360**		
(5) Growing‐degree days (GDD) until leaf expansion	0.079	0.018	−0.004	−0.042	**0.109**	
(6) GDD until flowering	0.033	**0.033**	−0.009	−0.377	**0.093**	**0.155**

Significant estimates are in bold.

Many studies have predicted strong plastic changes in phenology in response to expected climate change (Kramer 1995; Anderson et al. 2012; Scheepens and Stöcklin 2013). Indeed, we also detected highly plastic responses in phenology and leaf size in a previous transplant experiment with *S. herbacea* (Sedlacek et al. 2015). Therefore, we conclude that *S. herbacea* possesses both high plasticity and an adaptive evolutionary potential through heritable variation for morphological and phenological traits related to performance that may allow the species to persist in situ through ongoing climate change, at least initially. However, it remains to be seen whether the pace of environmental change exceeds the speed of possible evolutionary responses in this species.

## Data Accessibility

- Microsatellite genotypes: Dryad
- Phenotypic data: Dryad
- Sampling locations are uploaded as Table in the Supporting Information.

## Conflict of Interest

None declared.

## Supporting information


**Figure S1.** Probability of identity (PI) for different combinations and numbers of SSR markers.
**Table S1.** Summary information of locations and environmental conditions of the 12 study sites.
**Table S2.** Mean day of snowmelt (Julian days), mean summer temperature (for August) and growing degree days (GDD) until the end of September for ridge and snowbed sites.
**Table S3**. Summary information for the seven SSR loci in the study on *Salix herbacea*.
**Table S4.** Summary information (mean, range, variance and actual variance or relatedness (r)) for the four relatedness estimators Lynch and Ritland (1999, LR), Queller and Goodnight (1989, QG), Wang (2002, WA) and Li et al. (1993, LI).
**Table S5.** Estimates of narrow‐sense heritability (*h* ^2^) and its 95% confidence intervals (lowCI, upCI), and estimates of the additive genetic variance (Va) and the residual variance (Vr) from multivariate animal models for leaf size, performance traits (change in stem number, proportion flowering stems) and phenological traits (snowmelt‐to‐leaf‐expansion interval, GDD until leaf expansion, GDD until flowering).
**Table S6**. Regression coefficient estimates (Est.) for ridge and snowbed microhabitats, separately.
**Table S7.** The matrix of additive genetic variances and covariances (G matrix) using the Queller and Goodnight (1989) relatedness estimator.
**Table S8.** The matrix of additive genetic variances and covariances (G matrix) using the Wang (2002) relatedness estimator.
**Table S9.** The matrix of additive genetic variances and covariances (G matrix) using the Li et al. (1993) relatedness estimator.
