# Supplementary material for: Evolutionary potential in the Alpine: trait heritabilities and performance variation of the dwarf willow Salix herbacea from different elevations and microhabitats
Source: Ecol Evol. 2016 May 12;6(12):3940–52. doi: 10.1002/ece3.2171 (PMC4972222; doi:10.1002/ece3.2171)
Supplement: Supplementary file 1 — Figure S1. Probability of identity (PI) for different combinations and numbers of SSR markers. Table S1. Summary information of locations and environmental conditions of the 12 study sites. Table S2. Mean day of snowmelt (Julian days), mean summer temperature (for August) and growing degree days (GDD) until the end of September for ridge and snowbed sites. Table S3. Summary information for the seven SSR loci in the study on Salix herbacea. Table S4. Summary information (mean, range, variance and actual variance or relatedness (r)) for the four relatedness estimators Lynch and Ritland (1999, LR), Queller and Goodnight (1989, QG), Wang (2002, WA) and Li et al. (1993, LI). Table S5. Estimates of narrow‐sense heritability (h 2) and its 95% confidence intervals (lowCI, upCI), and estimates of the additive genetic variance (Va) and the residual variance (Vr) from multivariate animal models for leaf size, performance traits (change in stem number, proportion flowering stems) and phenological traits (snowmelt‐to‐leaf‐expansion interval, GDD until leaf expansion, GDD until flowering). Table S6. Regression coefficient estimates (Est.) for ridge and snowbed microhabitats, separately. Table S7. The matrix of additive genetic variances and covariances (G matrix) using the Queller and Goodnight (1989) relatedness estimator. Table S8. The matrix of additive genetic variances and covariances (G matrix) using the Wang (2002) relatedness estimator. Table S9. The matrix of additive genetic variances and covariances (G matrix) using the Li et al. (1993) relatedness estimator. [file ECE3-6-3940-s001.docx]

**Supplementary Figures and Tables**

In support of the article “Evolutionary potential in the Alpine: trait heritabilities and performance variation of the dwarf willow *Salix herbacea* from different elevations and microhabitats” by Janosch Sedlacek, Andrés Cortés, Julia Wheeler, Oliver Bossdorf, Guenter Hoch, [Jaroslav Klápště](http://www.researchgate.net/profile/Jaroslav_Klapst), Christian Lexer, Christian Rixen, Sonja Wipf, Sophie Karrenberg and Mark van Kleunen.

**
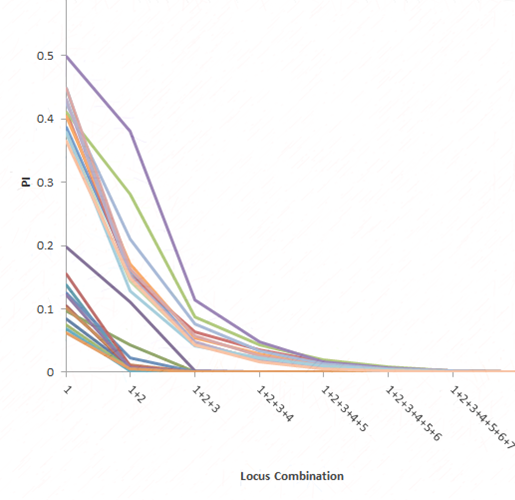
**

**Figure S1.** Probability of identity (PI) for different combinations and numbers of SSR markers.

**Table S1.** Summary information of locations and environmental conditions of the 12 study sites. Geographical coordinates (latitude, longitude), elevation, number of *Salix herbacea* patches sampled within each site, and the across-year (2011-2013) means of the Julian day of snowmelt, of the mean summer temperature (for August) and of the growing degree days (GDD) until the end of the season (end of September). Site abbreviations follow a combination of the first letter of the respective mountain (J = Jakobshorn, S = Schwarzhorn, W = Wannengrat), elevation (H = high, L = low) and microhabitat (R = ridge, S = snowbed).

| **Site** | **Latitude (°)** | **Longitude (°)** | **Elevation [m]** | **No. patches** | **Mean day of snowmelt** | **Mean summer temperature [°C]** | **GDD until end of season** |
| --- | --- | --- | --- | --- | --- | --- | --- |
| JHR | 46.7732761 | 9.85241808 | 2445 | 64 | 103 | 15.7 | 315 |
| JHS | 46.7725085 | 9.85312839 | 2415 | 71 | 158 | 12.6 | 161 |
| JLR | 46.7758917 | 9.86083274 | 2181 | 72 | 134 | 13.1 | 809 |
| JLS | 46.7730008 | 9.86376088 | 2215 | 65 | 136 | 12.6 | 641 |
| SHR | 46.732154 | 9.94912844 | 2768 | 80 | 141 | 9.7 | 205 |
| SHS | 46.7319127 | 9.95083011 | 2704 | 78 | 164 | 8.3 | 78 |
| SLR | 46.7418781 | 9.96367239 | 2350 | 68 | 131 | 13.7 | 146 |
| SLS | 46.7417032 | 9.96375766 | 2349 | 69 | 173 | 13.7 | 275 |
| WHR | 46.7915815 | 9.76818204 | 2616 | 67 | 133 | 7.5 | 187 |
| WHS | 46.7909068 | 9.76821916 | 2625 | 79 | 181 | 8.0 | 157 |
| WLR | 46.799941 | 9.78172517 | 2353 | 72 | 127 | 12.0 | 475 |
| WLS | 46.7999726 | 9.7823031 | 2333 | 85 | 191 | 11.5 | 238 |

**Table S2.** Mean day of snowmelt (Julian days), mean summer temperature (for August) and growing degree days (GDD) until the end of September for ridge and snowbed sites. Differences in means were tested using paired t-tests (df = 5).

| **Trait** | **Ridge (mean ± SE)** | **Snowbed (mean ± SE)** | **t** | **p-value** |
| --- | --- | --- | --- | --- |
| Mean day of snowmelt | 131.25 ± 1.26 | 167.17 ± 7.87 | -4.192 | 0.009 |
| Mean summer temperature [°C] | 11.94 ± 1.21 | 11.12 ± 0.98 | 1.567 | 0.178 |
| GDD until end of season | 356.02 ± 102.71 | 258.32 ± 81.51 | 1.842 | 0.125 |

**Table S3**. Summary information for the seven SSR loci in the study on *Salix herbacea*. For each locus, the table gives the names of the fluorescent dyes (Fl. Dye), the number of genotypes (N), the number of alleles, the size of the smallest and largest alleles, the range of allele frequencies, the observed (HO) and expected (HE) heterozygosity, the effective number of alleles (AE = 1/(1-HE)), the estimated frequency of null alleles and Chi^2^ test statistics of tests for deviations from Hardy-Weinberg equilibrium (*: p<0.001). Superscript numbers above each locus indicate which loci were used in the first (1) and the second (2) multiplex PCR. Due to interference with another marker, an additional eight marker from the second multiplex run was excluded.

| **Locus** | **Fl. dye** | **N** | **No. alleles** | **Smallest (bp)** | **Largest (bp)** | **Range of allele frequencies** | **HO** | **HE** | **AE** | **freq. Null alleles** | **HWE df** | **HWE Chi^2^** |
| --- | --- | --- | --- | --- | --- | --- | --- | --- | --- | --- | --- | --- |
| ASP112322^1^ | NED | 863 | 14 | 132 | 159 | 0.008 - 0.415 | 0.618 | 0.751 | 4.013 | 0.0854 | 91 | 466.052* |
| gSIMCT024^1^ | VIC | 919 | 29 | 274 | 323 | 0.001 - 0.169 | 0.832 | 0.916 | 11.923 | 0.0453 | 406 | 2312.572* |
| gSIMCT035^1^ | FAM | 850 | 10 | 283 | 299 | 0.003 - 0.472 | 0.369 | 0.699 | 3.326 | 0.202 | 45 | 1293.585* |
| GCPM_1255^1^ | FAM | 935 | 15 | 189 | 212 | 0.001 - 0.395 | 0.522 | 0.780 | 4.551 | 0.1481 | 105 | 2257.863* |
| GCPM_1812^2^ | VIC | 936 | 16 | 181 | 213 | 0.001 - 0.358 | 0.420 | 0.791 | 4.787 | 0.2229 | 120 | 4223.61* |
| ORPM_312^2^ | PET | 798 | 23 | 183 | 227 | 0.005 - 0.159 | 0.519 | 0.906 | 10.682 | 0.2124 | 253 | 3753.959* |
| gSIMCT052^2^ | NED | 883 | 56 | 274 | 385 | 0.001 - 0.074 | 0.605 | 0.965 | 28.277 | 0.1844 | 1540 | 8825.979* |
| multilocus |  | 883.4 | 23.3 |  |  |  | 0.555 | 0.830 | 9.651 |  |  |  |

**Table S4.** Summary information (mean, range, variance and actual variance or relatedness (r)) for the four relatedness estimators Lynch & Ritland (1999, LR), Queller & Goodnight (1989, QG), Wang (2002, WA) and Li *et al.* (1993, LI). Standard errors were calculated by jackknifing across loci.

| **Estimator** | **Mean (r) ± SE** | **Range of r** | **Var (r)** | **Actual var (r) ± SE** |
| --- | --- | --- | --- | --- |
| LR | -0.0009 ± 0.0007 | -0.523 – 0.995 | 0.0108749 | 0.0011 ± 0.0007 |
| QG | -0.0021 ± 0.0009 | -0.620 – 0.946 | 0.0332287 | 0.0041 ± 0.0023 |
| WA | -0.0483 ± 0.0257 | -0.698 – 0.965 | 0.0194501 | 0.0033 ± 0.0032 |
| LI | -0.0235 ± 0.0209 | -0.693 – 0.983 | 0.0244375 | 0.0028 ± 0.0033 |

**Table S5.** Estimates of narrow-sense heritability (*h^2^*) and its 95% confidence intervals (lowCI, upCI), and estimates of the additive genetic variance (Va) and the residual variance (Vr) from multivariate animal models for leaf size, performance traits (change in stem number, proportion flowering stems) and phenological traits (snowmelt-to-leaf-expansion interval, GDD until leaf expansion, GDD until flowering). Shown are the results based on the Lynch & Ritland (1999, LR), Queller & Goodnight (1989, QG), Wang (2002, WA) and Li *et al.* (1993, LI) relatedness estimators.

| **Trait** | **Relatedness estimator** | **h^2^** | **lowCI** | **upCI** | **Va** | **Vr** |
| --- | --- | --- | --- | --- | --- | --- |
| Leaf area | LR | 0.061 | 0.011 | 0.112 | 0.128 | 1.952 |
|  | QG | 0.063 | 0.013 | 0.113 | 0.130 | 1.944 |
|  | WA | 0.139 | 0.044 | 0.234 | 0.312 | 1.933 |
|  | LI | 0.075 | 0.022 | 0.128 | 0.156 | 1.929 |
| Change in stem number | LR | 0.071 | 0.014 | 0.128 | 0.016 | 0.207 |
|  | QG | 0.063 | 0.007 | 0.120 | 0.014 | 0.209 |
|  | WA | 0.120 | 0.028 | 0.213 | 0.029 | 0.209 |
|  | LI | 0.062 | 0.008 | 0.115 | 0.014 | 0.210 |
| Proportion flowering stems | LR | 0.034 | -0.018 | 0.087 | 0.001 | 0.036 |
|  | QG | 0.060 | 0.003 | 0.118 | 0.002 | 0.035 |
|  | WA | 0.025 | -0.018 | 0.068 | 0.001 | 0.037 |
|  | LI | 0.026 | -0.017 | 0.068 | 0.037 | 0.037 |
| Snowmelt-to-leaf-expansion interval | LR | 0.178 | 0.111 | 0.245 | 10.360 | 47.842 |
|  | QG | 0.218 | 0.139 | 0.297 | 12.936 | 46.485 |
|  | WA | 0.266 | 0.169 | 0.363 | 17.534 | 48.302 |
|  | LI | 0.212 | 0.130 | 0.294 | 12.927 | 48.051 |
| GDD until  leaf expansion | LR | 0.141 | 0.008 | 0.275 | 0.109 | 0.664 |
|  | QG | 0.133 | 0.006 | 0.260 | 0.101 | 0.662 |
|  | WA | 0.026 | -0.064 | 0.117 | 0.020 | 0.741 |
|  | LI | 0.134 | 0.013 | 0.255 | 0.104 | 0.677 |
| GDD until flowering | LR | 0.181 | 0.032 | 0.330 | 0.155 | 0.700 |
|  | QG | 0.145 | 0.006 | 0.284 | 0.122 | 0.718 |
|  | WA | 0.392 | 0.179 | 0.605 | 0.433 | 0.671 |
|  | LI | 0.151 | 0.020 | 0.282 | 0.129 | 0.727 |

**Table S6**. Regression coefficient estimates (Est.) for ridge and snowbed microhabitats, separately. Linear mixed models were run separately for the response variables proportion of flowering stems and change in stem number, and the explanatory variables included leaf size, snowmelt-to-leaf-expansion interval, GDD until leaf expansion and GDD until flowering, with plot nested within transect as random effects.

|  | |  | **Ridge** | | |  | **Snowbed** | | |
| --- | --- | --- | --- | --- | --- | --- | --- | --- | --- |
| **Relative fitness** | **Trait (standardized)** |  | **Est.** | **t** | **p** |  | **Est.** | **t** | **p** |
| Proportion flowering stems | Leaf size |  | -0.005 | -0.041 | 0.967 |  | 0.034 | 0.263 | 0.794 |
|  | Snowmelt-to-leaf-expansion interval |  | 0.014 | 0.093 | 0.926 |  | 0.180 | 1.308 | 0.195 |
|  | GDD until leaf expansion |  | -0.031 | -0.237 | 0.813 |  | -0.136 | -1.106 | 0.273 |
|  | GDD until flowering |  | 0.232 | 1.722 | 0.090 |  | 0.153 | 1.255 | 0.214 |
| Change in stem number | Leaf size |  | -5.165 | -3.272 | 0.001 |  | -1.406 | -0.844 | 0.400 |
|  | Snowmelt-to-leaf-expansion interval |  | -3.488 | -2.211 | 0.029 |  | 0.731 | 0.307 | 0.759 |
|  | GDD until leaf expansion |  | -3.804 | -2.348 | 0.021 |  | -2.057 | -1.216 | 0.227 |
|  | GDD until flowering |  | 2.481 | 1.445 | 0.151 |  | -2.512 | -1.623 | 0.107 |

**Table S7.** The matrix of additive genetic variances and covariances (G matrix) using the Queller & Goodnight (1989) relatedness estimator. Significant estimates are in bold.

| Trait | (1) | (2) | (3) | (4) | (5) | (6) |
| --- | --- | --- | --- | --- | --- | --- |
| (1) Leaf size | **0.312** |  |  |  |  |  |
| (2) Change in stem number | 0.014 | **0.029** |  |  |  |  |
| (3) Proportion of flowering stems | 0.006 | 0.001 | 0.001 |  |  |  |
| (4)Snowmelt-to-leaf-expansion interval | -0.039 | -0.008 | -0.131 | **17.534** |  |  |
| (5) GDD until leaf expansion | 0.014 | 0.001 | -0.001 | 0.186 | 0.020 |  |
| (6) GDD until flowering | 0.012 | 0.020 | -0.010 | -0.193 | 0.031 | **0.433** |

**Table S8.** The matrix of additive genetic variances and covariances (G matrix) using the Wang (2002) relatedness estimator. Significant estimates are in bold.

| Trait | (1) | (2) | (3) | (4) | (5) | (6) |
| --- | --- | --- | --- | --- | --- | --- |
| (1) Leaf size | **0.130** |  |  |  |  |  |
| (2) Change in stem number | 0.013 | **0.014** |  |  |  |  |
| (3) Proportion of flowering stems | 0.008 | 0.003 | 0.002 |  |  |  |
| (4)Snowmelt-to-leaf-expansion interval | 0.034 | 0.063 | -0.098 | **12.936** |  |  |
| (5) GDD until leaf expansion | 0.058 | 0.003 | 0.000 | 0.034 | 0.101 |  |
| (6) GDD until flowering | 0.019 | 0.017 | -0.007 | -0.056 | 0.079 | 0.122 |

**Table S9.** The matrix of additive genetic variances and covariances (G matrix) using the Li *et al.* (1993) relatedness estimator. Significant estimates are in bold.

| Trait | (1) | (2) | (3) | (4) | (5) | (6) |
| --- | --- | --- | --- | --- | --- | --- |
| (1) Leaf size | **0.156** |  |  |  |  |  |
| (2) Change in stem number | 0.019 | **0.014** |  |  |  |  |
| (3) Proportion of flowering stems | 0.007 | 0.001 | 0.037 |  |  |  |
| (4)Snowmelt-to-leaf-expansion interval | -0.078 | 0.002 | -0.085 | **12.927** |  |  |
| (5) GDD until leaf expansion | 0.077 | 0.012 | 0.001 | -0.113 | **0.104** |  |
| (6) GDD until flowering | 0.038 | 0.021 | -0.009 | -0.251 | **0.100** | **0.129** |
